# Measurement of Scenic Spots Sustainable Capacity Based on PCA-Entropy TOPSIS: A Case Study from 30 Provinces, China

**DOI:** 10.3390/ijerph15010010

**Published:** 2017-12-22

**Authors:** Xuedong Liang, Canmian Liu, Zhi Li

**Affiliations:** 1 The Economy and Enterprise Development Institute, Sichuan University, Chengdu 610065, China; liangxuedong@scu.edu.cn; 2 Business School, Sichuan University, Chengdu 610065, China; 2016225025022@stu.scu.edu.cn

**Keywords:** sustainable development of scenic spots, principal components analysis, entropy, TOPSIS, sustainability

## Abstract

In connection with the sustainable development of scenic spots, this paper, with consideration of resource conditions, economic benefits, auxiliary industry scale and ecological environment, establishes a comprehensive measurement model of the sustainable capacity of scenic spots; optimizes the index system by principal components analysis to extract principal components; assigns the weight of principal components by entropy method; analyzes the sustainable capacity of scenic spots in each province of China comprehensively in combination with TOPSIS method and finally puts forward suggestions aid decision-making. According to the study, this method provides an effective reference for the study of the sustainable development of scenic spots and is very significant for considering the sustainable development of scenic spots and auxiliary industries to establish specific and scientific countermeasures for improvement.

## 1. Introduction

The sustainable development of tourism occupies a dominant position in Sustainable Development Goals (SDGs) of the whole world at present. In the opinion of the World Tourism Organization (WTO), sustainable development of tourism should not only meet existing demands from scenic spots and tourists but also meet future ones [1]. A scenic spot is the spatial carrier of all tourism activities, a place focusing on tourism and relevant actions and an independently administered area possessing corresponding tourism facilities and providing corresponding tourism services. More and more weaknesses and problems have appeared along with the prosperous development of the tourism industry, including more difficult management of scenic spots, increased environmental pressure and serious waste of resources brought by the rapid growth of tourism and excessive development of tourism resources. Many scholars and even the Central Government are focusing on achieving a balance between the development of tourism economy and resources; therefore, sustainable development of scenic spots emerges to be an important area in the study on the tourism development. For the purpose of promoting the sustainable development of tourism, sustainability of existing actions and measures must be evaluated to measure and investigate the sustainable development of scenic spots effectively. As a result, this paper starts from preliminary achievements to conduct the multiple-objective study on the sustainable capacity of Chinese scenic spots; establishes the measurement index system with comprehensive consideration of influential factors of tourism economy, society, resources and environment; establishes the sustainable capacity measurement model of regional tourism by PCA-entropy TOPSIS method; carries out empirical study on the sustainable development of Chinese scenic spots; and provides a reference for strengthening the competitiveness and improving the sustainable capacity of scenic areas.

## 2. Literature Review

### 2.1. Research on the Development of Scenic Spots

In order to promote the sustainable development of scenic spots, scholars have undertaken extensive and in-depth exploration from various perspectives, mainly including those described below.

The first is to discuss about the influence of a certain factor or driving mechanism on the sustainable development of scenic spots. Jin and Hu start from the crowding of scenic spots to study the influence of crowding as perceived by tourists on the development of scenic spots, focus on analyzing the psychological influence and provide suggestions to the management of scenic spots [2]. Yao and Ma established a shunt and scheduling model of tourists and adopt the optimal shunt scheme to facilitate the compound balance of scenic spots to relieve the conflict between economic development and environmental protection [3]. Duan and Li put forward a dynamic pricing model from the perspective of economic development of scenic spots and improve the revenue management of scenic spots by scientific and reasonable pricing strategy and price system [4]. Iniesta-Bonillo et al. discuss the sustainability of scenic spots perceived by tourists and the relation between perceived value and satisfaction of tourists, recognizing that sustainability is a multi-level structure consisting of economy, social culture and environment levels [5]. Liao et al. established the SD model promoting the sustainable development of scenic spots on the basis of economic and environmental subsystems of scenic spots to assist scenic spots in tackling the conflict between tourism development and ecological environment, and finally test and verify the effectiveness of such model by analyzing the case of Jiuzhaigou [6]. Cucculelli and Goffi introduced sustainability indexes to the competitiveness model of scenic spots and inspect the empirical effect of such model by principal components analysis and regression analysis, proving the critical role of variables of sustainability in the competitiveness of scenic spots [7].

The second is to discuss about the influence of coordinated development of stakeholders on the sustainable development of scenic spots. Mónica et al. evaluated the sustainable tourism strategy of stakeholders of National Park by analytic network analysis and Delphi-type judgment-ensuring process [8]. Carlos-Rosell and Mäkinen introduced the theoretical and methodological frame attracting partners from tourism organizations and stakeholders in the tourism sustainability evaluation [9]. Xu et al. start from the adoption by social media to study the influence of adoption by social media on operational efficiency of scenic spots by three-stage DEA model and discover that the technical efficiency of most Chinese scenic spots is quite low due to various kinds of adoption by social media [10]. Begum et al. studied whether the government, local citizen and private entrepreneur have different opinions in the sustainable development of scenic spots and discover that the government, individual and local community play an important role in achieving the sustainable development of scenic spots [11].

The third is to organize the multiple-objective study and evaluation of the development of scenic spots to facilitate the sustainable development of scenic spots. Early in 1998, Garrod and Fyall pointed out that the focus should be shifted from the definition to the practice of sustainable development of tourism, and established a frame to measure the sustainable tourism [12]. Evaluation indexes of sustainable development tourism and the corresponding application are being progressed while the connotation of sustainable development tourism is understood better and better. Various planning frames, including bearing capacity, acceptable variation range, preference and experience of tourists, life cycle of destination, comfort indexes and tourist influence management, are applied to evaluation indexes of sustainable development. Most scholars classify indexes by establishing the index system but much information is still overlapped or missed. With respect to the evaluation of sustainable development of scenic spots, Gössling et al. put forward the ecological footprint analysis to access the tourism sustainability by comparing the ecological footprint and ecological capacity of tourism [13]. Lee and Hsieh determined key dimensions and indexes of the sustainable wetland tourism by fuzzy Delphi method, verifies the relative weight of such dimensions and indexes by AHP and manages the sustainable wetland tourism by such indexes [14]. Zhang also established the evaluation model of sustainable development of ecological scenic spots by a similar method [15].

### 2.2. Summary on Previous Research

Significant achievements have been made with respect to the study of sustainable development of scenic spots and many scholars have made great contributions resulting from their own understanding of the nature of sustainable scenic spots. However, there are few quantitative studies on the sustainable development of scenic spots. Existing ones often focus on a certain aspect of the scenic spot development, such as natural resource [16], ecological environment [17] and industrial development [18]; and most of them only concern a small area, such as a certain province [19], a certain county [15,20] or a certain scenic spot [16,17]. Moreover, it has to be stressed that analytic hierarchy process [14,15], Delphi method [8], ecological footprint method [13,21] and principal components analysis [7,18] are adopted the most frequently in the measurement of sustainable development of scenic spots, but all of them are somewhat defective. Analytic hierarchy processes and the Delphi method assign the weight of indexes based on existing work experience and knowledge of the expert or researcher that can be quite subjective, often exaggerating or minimizing the effect of some indexes which somewhat affects the scientific nature of the results; although the ecological footprint method measures the influence of human beings on the natural ecological system, it seldom considers social and economic development and neglects the strength of regional feature and ecological footprint, and the index data is difficult to obtain; principal components analysis is defective that it stacks the expressed value of several principal components influencing the sustainable development of scenic spots simply in a linear manner but does not consider the coordination among various elements of the sustainability system, thus being difficult to reflect the sustainability of the entire system. In view of such weaknesses, this paper puts forward a principal component-entropy TOPSIS-based measurement model of sustainable development of scenic spots. Firstly, it relies on the actual situation of each province and city of China to consider elements of economy, society, resource and environment in relation to the sustainable development of scenic spots to establish a more comprehensive measurement index system; secondly, it puts forward the measurement model of sustainable development of scenic spots. Such model optimizes the measurement index system by principal components analysis, determines the weight of principal components by entropy method, and computes the value and rank of sustainable development of scenic spots of each province and city by TOPSIS method, and finally proposes suggested countermeasures to improve the sustainable development on the basis of actual situation of scenic spots of each province and city.

## 3. Summary of Sustainable Capacity Measurement Mode 

### 3.1. Measurement Model

**Figure 1:**
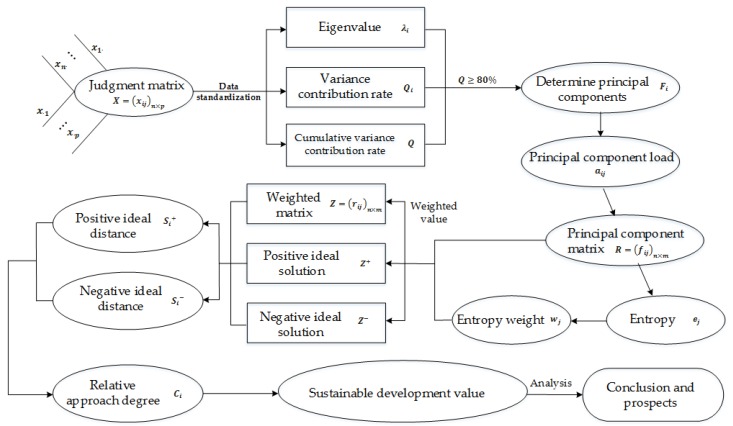
Measurement Model.

Following the logic shown in Figure 1, this paper integrates principal components analysis, entropy method and TOPSIS method to establish the measurement model of sustainable capacity of tourism, in which, principal components analysis relies on the raw data of measurement indexes to extract principal components influencing the sustainable development of scenic spots to optimize the measurement index system; entropy method assigns the objective weight to each principal component extracted by the principal components analysis; and TOPSIS method relies on the distance between the weighted score and the ideal solution of principal component of each measured object (province and city), so as to obtain the sustainable capacity and rank of each scenic spot.

In Figure 1, $x_{1j}$~$x_{np}$ represent the raw data of tourism sustainability of each province and city, *R* represents principal component score matrix after extraction and rotation of factors.

### 3.2. Principal Components Analysis

Principal Components Analysis (PCA) reduces the dimensionality of a higher dimensional variable space and replaces existing multi-dimensional variables with a few aggregate variables by linear transformation and abandoning some information while minimizing the loss of raw data [22]. Its basic concept is that assuming there are *n* measured objects and each object possesses *p* index data to constitute the initial measurement matrix of n×p. Generally speaking, there must be certain linear relation among such *p* indexes, so it is able to obtain m aggregate indexes from *p* indexes (m≤p) by linear algebra to replace such *p* indexes by such *m* indexes with little information lost [18]. This paper extracts principal components influencing the sustainable development of scenic spots by the principal components analysis, assumes $F_{i}$ to be the principal component and assumes $x_{i}$ to be the original measurement index. The computation steps are listed below:

(1) Data standardization. This paper relies on the raw data of measurement index of each province and city to establish the initial measurement matrix $X={\left(x_{ij}\right)}_{n\times p}$. In order to eliminate the difference of selected indexes in the quantity and size, collected raw data of indexes need to be standardized to obtain the standardized matrix Z;
(2) Computing related coefficient matrix $R={\left(r_{ij}\right)}_{p\times p}$;
(3) According to the characteristic equation |λE−R|=0, computing that characteristic value is $\lambda_{i}\left(i=1,2,\ldots ,p\right)$ and characteristic vector is $e_{i}\left(i=1,2,\ldots ,p\right)$;
(4) Computing principal component contribution rate $Q_{i}$ and accumulated contribution rate Q:
  (1) $$Q_{i}=\frac{\lambda_{i}}{\sum_{k=1}^{p}\lambda_{k}}\left(i=1,2,\ldots ,p\right)$$
  (2) $$Q=\frac{\sum_{k=1}^{i}\lambda_{k}}{\sum_{k=1}^{p}\lambda_{k}}\left(i=1,2,\ldots ,p\right)$$

If the accumulated contribution rate of the first *i* principal components has reached 80–95%, the first *i* principal components should be set as new variables.

(5) Computing principal component loading $a_{ij}$:
  (3) $$a_{ij}=\sqrt{\lambda_{i}}e_{ij}\left(i,j=1,2,\ldots ,p\right)$$
(6) Computing the score of each principal component:
  (4) $$\{\begin{array}{c} F_{1}=a_{11}x_{1}+a_{12}x_{2}+a_{13}x_{3}+\cdots +a_{1p}x_{p}\\ F_{2}=a_{21}x_{1}+a_{22}x_{2}+a_{23}x_{3}+\cdots +a_{2p}\\ F_{3}=a_{31}x_{1}+a_{32}x_{2}+a_{33}x_{3}+\cdots +a_{3p}x_{p}\\ \ldots \;\ldots \;\ldots \\ F_{m}=a_{m1}x_{1}+a_{m2}x_{2}+a_{m3}x_{3}+\cdots +a_{mp}x_{p} \end{array}$$

### 3.3. Entropy Method Based TOPSIS Analysis

#### 3.3.1. Determination of Entropy of Principal Component Factor

The entropy method is an objective method for constructing the judgment matrix based on the value of evaluation index and determining the weight by the degree of variation of each index. Determining the weight of each principal component by entropy method may eliminate the influence brought by subjective factors to the largest extent to obtain a more practical result. Steps of determining the entropy weight are listed below:

(1) Standardization of principal component indexes

Assuming there are n measured objects (province and city) and m principal component factors, the standardization matrix established according to the score of each principal component of the measured object is $R={\left(f_{ij}\right)}_{n\times m}\left(i=1,2,\cdots ,n;j=1,2,\cdots ,m\right)$.

(2) Determination of entropy and entropy weight of principal component factors

In accordance with the definition of entropy, the entropy value $e_{j}$ and entropy weight $w_{j}$ of the *j*th principal component factor are:

(5) $$e_{j}=-\frac{1}{lnn}\overset{n}{\underset{i=1}{\sum }}\left[b_{ij}lnb_{ij}\right]$$

(6) $$b_{ij}=\frac{f_{ij}+1}{\sum_{i=1}^{n}\left(f_{ij}+1\right)}$$

(7) $$w_{j}=\frac{1-e_{j}}{\sum_{j=1}^{m}\left(1-e_{j}\right)}$$

In the equation, the original expression of $b_{ij}$ should be $b_{ij}=\frac{f_{ij}}{\sum_{i=1}^{n}f_{ij}}$. When $b_{ij}$ = 0, $lnb_{ij}$ is meaningless [23], so the initial expression should be revised to Equation (6).

#### 3.3.2. Determination of Approach Degree by TOPSIS Method

Technique for Order Preference by Similarity to an Ideal Solution (“TOPSIS” for short), which is the one of group of MCDM methods, was first proposed by Hwang and Yoon in 1981 [24]. TOPSIS is usually utilized in definite schemes of the system engineering to analyze the objective decision [18]. Core concept: The optimal scheme should be the closest to the positive ideal scheme and the farthest from the negative ideal scheme. This model can objectively and comprehensively reflect the level of sustainable development by calculating the closeness degree between an evaluation value and its ideal solution [25]. Specific steps are as follows:

(1) Establishing weighted standardized matrix $Z={\left(r_{ij}\right)}_{n\times m}$ to determine positive ideal solution $Z^{+}$ and negative ideal solution $Z^{-}$:

(8) $$Z_{ij}={\left(r_{ij}\right)}_{n\times m}={\left(w_{j}y_{ij}\right)}_{n\times m}=\left[\begin{array}{cccc} w_{1}y_{11} & w_{2}y_{12} & \cdots & w_{m}y_{1m}\\ w_{1}y_{21} & w_{2}y_{22} & \cdots & w_{m}y_{2m}\\ \vdots & \vdots & \ddots & \vdots \\ w_{1}y_{n1} & w_{2}y_{n2} & \cdots & w_{m}y_{nm} \end{array}\right]$$

In the equation: $w_{j}$ is the entropy weight of the *j*th principal component factor and *y_ij_* is the score of the *j*th principal component of the *i*th measured object:

(9) $$\{\begin{array}{c} Z^{+}=\left\{\left(\left(maxZ_{ij}|j\in J_{1}\right),\left(minZ_{ij}|j\in J_{2}\right)|i=1,2,\cdots ,m\right)\right\}=\left\{z_{1}^{+},z_{2}^{+},\cdots ,z_{n}^{+}\right\}\\ Z^{-}=\left\{\left(\left(maxZ_{ij}|j\in J_{2}\right),\left(minZ_{ij}|j\in J_{1}\right)|i=1,2,\cdots ,m\right)\right\}=\left\{z_{1}^{-},z_{2}^{-},\cdots ,z_{n}^{-}\right\} \end{array}$$

In the equation: $J_{1}$ is the positive index of the *j*th index and $J_{2}$ is the *j*th inverse index.

(2) Computing the distance of each province and city to the positive ideal point ${S_{i}}^{+}$ and to the negative ideal point ${S_{i}}^{-}$ to obtain the relative approach of each province and city to the ideal objective $C_{i}$ to demonstrate the sustainable capacity of each province and city:

(10) $$\{\begin{array}{c} {S_{i}}^{+}=\sqrt{\overset{n}{\underset{j=1}{\sum }}{\left[Z_{ij}-Z_{j}^{+}\right]}^{2}}\\ {S_{i}}^{-}=\sqrt{\overset{n}{\underset{j=1}{\sum }}{\left[Z_{ij}-Z_{j}^{-}\right]}^{2}} \end{array}\;i=1,2,\cdots ,m$$

(11) $$C_{i}=\frac{{S_{i}}^{-}}{{S_{i}}^{+}+{S_{i}}^{-}}\;i=1,2,\cdots ,m$$

A larger $C_{i}$ indicates a stronger sustainable capacity of the area, and vice versa.

## 4. Establishment of Measurement Index System of Sustainable Development

Development of scenic spots more and more involves challenges from society, economy and the ecological environment to complement the urgently needed sustainable development. The sustainability of scenic spots is affected by many factors, commonly comprising economic, resource and environmental aspects. The essence of sustainable development of scenic spots is to ensure the long-term reasonable economic development and fair distribution of social and economic benefits to stakeholders (auxiliary industries in relation to the sustainable development of scenic spots); and resources and environment should be utilized to the optimal extent as critical elements for the development of scenic spots. In the establishment of measurement indexes of sustainable development of scenic spots, indexes should not only reflect the authentic situation of sustainable tourism but also keep sufficient comparability and applicability for a certain period. Therefore, based on the existing relevant literature [15,18,20,26,27,28,29,30,31,32,33,34,35,36,37], this paper establishes the measurement index system of sustainable development sustainability of scenic spots from perspectives of the self-sustainability and auxiliary industry of scenic spots. Self-sustainability demonstrates the resource condition, economic benefit and ecological environment of scenic spots while auxiliary industry sustainability demonstrates the development of stakeholders of scenic spots, as shown in Table 1.

Such an index system of the sustainable development of scenic spots divides two types of sustainable development into five aspects, including resource condition, economic benefits, ecological environment, auxiliary industry scale and benefit (primary index), and establishes measurement indexes under such five aspects (secondary index). The logic of index selection is as follows.

Resource condition of scenic spot, including natural resource, human resource and market resource, is fundamental for the sustainable development. Natural resource is the foundation and precondition of tourism activity. Difference and relative advantage of natural resource are critical for the tourism activity and directly influence the selection and flow of tourists. Natural resource indicators include B11 and B12. Moreover, management of the scenic spot is only effective under the support from human resource, the situation of human resources in scenic spots is reflected by B13, and the market resources are measured by B14 and B15.

Economy of scenic spots, which is the economic benefit generated by tourism activities on the basis of resource condition, offers a physical guarantee of the sustainable development of the scenic spot. It comprises of economic benefits (reflected by B21–B24) from food & beverage, amusement and sightseeing, and also experience of tourists brought by tourism activities (measured by B25).

Ecological environment of scenic spot is the radical guarantee of the sustainable development. Ecological environment should be concerned at all times while tourism resources are being developed reasonably. Ecological environment is especially important when the green, low-carbon and cyclic economic development is advocated. Therefore, ecological environment indexes should cover the greening of scenic spot and ecological environment improvement. B31 and B34 quantitatively reveal the amount of atmospheric pollutant emissions and the number of treatment facilities caused by the development of tourism economy; B32 and B33 can demonstrate the situation of green cover of green space and forest around the scenic spot; B35 and B36 outline the status of pollutants and waste disposal.

Auxiliary industry refers to surrounding auxiliary industries and transportation industry, which are important parts of stakeholders of the development of scenic spot. While integrating its own resource advantages in the sustainable development, the scenic spot should give consideration to the development demand of stakeholders (auxiliary industry). On the other hand, coordination and cooperation of the auxiliary industry facilitates the sustainable development of the scenic spot greatly. Therefore, measurement of the sustainable capacity of the scenic spot should also include measurement indexes of the development of auxiliary industry. B41 and B43 measure the size of the scenic area auxiliary industry; B42 and B44 can better reflect the human resources situation of auxiliary industry in scenic spots; B45 and B46 reveal the training status of tourism professionals; B47 and B48 reflect the accessibility of scenic spots; B51–B56 are mainly used for the economic benefits of scenic auxiliary industry (including tourism enterprises and transportation).

**Figure 2:**
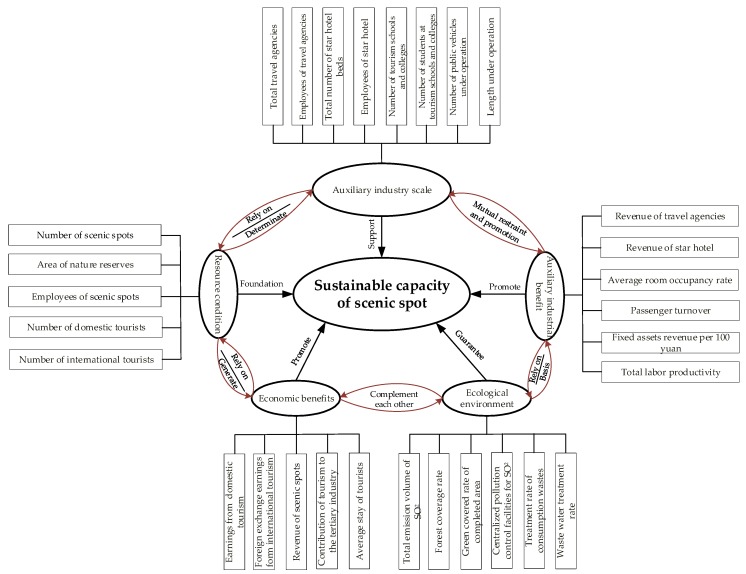
Indicator Logic Diagram.

It should be noted that these indexes are correlated and mutually promoted to drive the sustainable development of scenic spots. As a result, study on the sustainable capacity of scenic spots should consider each index comprehensively and conduct the analysis one by one. The relation among indexes and evaluation objectives of the sustainable capacity of scenic spots is shown in Figure 2.

## 5. Empirical Study

As required by the index system, the data in this study mainly comes from The Yearbook of China Tourism 2016 [38], China Statistical Yearbook 2016 [39], China Statistical Yearbook on Environment 2016 [40] and relevant statistical materials of each province and city of China. The data of some indexes is converted from the raw data through the computation formula. As there is missing value in the index of Tibet, this study does not involve Tibet. Relying on the principal component analysis-entropy TOPSIS method based measurement model, this paper conducts the following empirical analysis of the sustainable capacity of scenic spots in each province and city of China in 2015.

### 5.1. Optimization of Measurement Index System by PCA

This paper adopts SPSS 22.0 (SPSS Inc., Chicago, IL, USA) to standardize the measurement index data and adopts principal components analysis to optimize the sustainable capacity measurement index system. Specific steps are listed below.

#### 5.1.1. Determination of Quantity of Principal Components

As per the principle of keeping accumulated variance contribution rate above 80%, 5 principal components are extracted from 16 indexes of self-sustainability of the scenic spot and 3 principal components are extracted from 14 indexes of sustainable capacity of auxiliary industry. Containing 80.231% and 88.118% of information respectively in the measurement of sustainable capacity of scenic spot, they are able to interpret and express the raw index (as shown in Table 2 and Table 3).

#### 5.1.2. Determination of Standardized Principal Component Coefficient

Table 4 is the principal component score coefficient matrix computed according to Formula (3), based on which principal component Expression (12) representing the sustainable capacity of the scenic spot and principal component Expression (13) representing the sustainable capacity of the auxiliary industry may be obtained.

Expression of five principal components of self-sustainability:

(12) $$\{\begin{array}{l} F_{1}=-0.079x_{11}+0.062x_{12}+0.053x_{13}+\cdots -0.087x_{36}\\ F_{2}=0.324x_{11}+0.09x_{12}+0.196x_{13}+\cdots +0.092x_{36}\\ F_{3}=-0.018x_{11}-0.352x_{12}-0.063x_{13}+\cdots +0.277x_{36}\\ F_{4}=0.035x_{11}-0.144x_{12}+0.097x_{13}+\cdots -0.076x_{36}\\ F_{5}=-0.184x_{11}-0.198x_{12}-0.136x_{13}+\cdots +0.038x_{36} \end{array}$$

Expression of three principal components of the auxiliary industry:

(13) $$\{\begin{array}{l} F_{1}=0.879x_{41}+0.933x_{42}+0.902x_{43}+\cdots +0.453x_{56}\\ F_{2}=-0.151x_{41}+0.19x_{42}-0.091x_{43}+\cdots +0.812x_{56}\\ F_{3}=-0.155x_{41}-0.352x_{42}-0.063x_{43}+\cdots -0.086x_{56} \end{array}$$

### 5.2. Entropy TOPSIS Based Measurement of Sustainable Capacity of Scenic Spots

#### Computation of Principal Component Decision Matrix and Entropy of Each Principal Component

The principal component decision matrix may be obtained by substituting the standardized value of indexes of 30 provinces and cities to Expressions (12) and (13). Assigning the weight of each principal component $w_{j}\left(j=1,2,\cdots ,8\right)$ through Formulas (5)–(7) may obtain the principal component decision matrix and the entropy weight of each principal component, as shown in Table 5. 

Multiplying the score and entropy weight of each principal component may obtain the judgment matrix *Z* and further obtain the positive ideal solution *Z*^+^ and negative ideal solution *Z*^−^ (Table 6). Relative approach degree $C_{i}$ between the sustainable capacity and the ideal solution of scenic spots in each province and city may be obtained according to the formula and the result is listed in Table 7.

## 6. Results Analysis

### 6.1. Analysis of Sustainable Development of Scenic Spots of Chinese Provinces and Cities

For the purpose of understanding the sustainable development of scenic spots in each province and city in a more direct manner, this paper is based on values of sustainable capacity listed in Table 7 to divide the sustainable development of scenic spots in 30 Chinese provinces and cities into four types, namely, high sustainability, intermediate sustainability, low sustainability and non-sustainability by natural breaks [41], the specific division basis and explanation as shown in Table 8, and adopts ArcGIS 10.0 (Esri, Redlands, CA, USA) to generate the comparison chart of the self-sustainability, sustainable capacity of auxiliary industry and overall sustainable capacity of scenic spots in each province and city.

**Figure 3:**
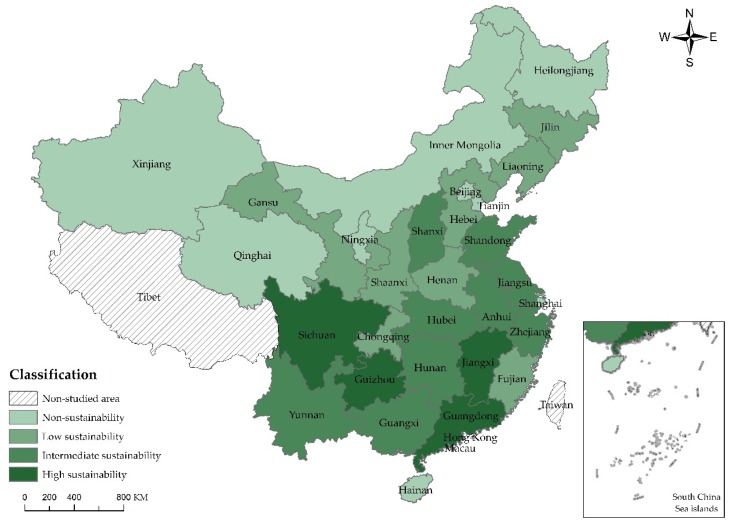
Comparison chart of self-sustainable capacity of scenic spots in China.

From the perspective of resource condition and economic benefit (self-sustainability) (Figure 3), areas with high sustainability include Guangdong (0.555) followed by Jiangxi (0.545), Sichuan (0.521) and Guizhou (0.516), where there are abundant natural tourism resources, favorable control of ecological environment and strong tourism market. Taking Guangdong for an example, it receives 743 million person-times of domestic tourists and 105,171,600 person-times of international tourists, possesses up to 103,087 management staff of scenic spot and 359 concentrated waste control facilities, much more than those in other areas. Areas with low sustainability include Qinghai (0.220), Ningxia (0.254) and Xinjiang (0.256) in the edge of China, where there are poor transportation, severe and bad ecological environment, insufficient tourism resources and low economic benefits from scenic spots. Taking Qinghai for an example, it receives only about 23 person-times of million domestic and international tourists with only CNY24.796 billion of revenue, much lower than CNY367.45, the average level of China. It also possesses poor ecological processing capability (sewage treatment rate is only 60%) and seriously damaged vegetation (forest coverage rate is only 5.63%). The sustainability of other areas are intermediate to low.

**Figure 4:**
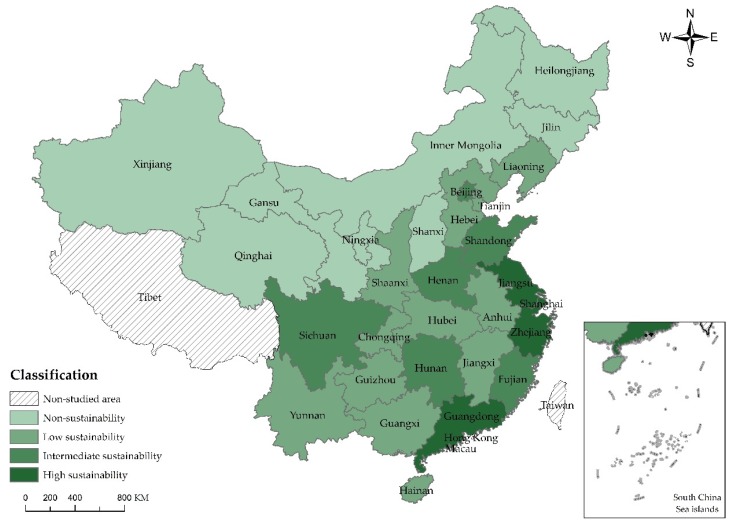
Comparison chart of sustainable capacity of auxiliary industry in China.

From the perspective of scale and benefit of auxiliary industry (Figure 4), areas with high sustainability include Jiangsu (0.625) followed by Guangdong (0.594). Zhejiang (0.572) and Shanghai (0.550). It can be easily noted that they are located in the east coastal area of China having enjoyed the policy support from the Chinese government for a long period that the tourism industry is highly mature with large scale, high benefit, rich human resources of tourism management, dense and convenient transportation network. Taking Zhejiang for an example, travel agencies in Zhejiang possess 24,066 staff and earn up to CNY25.853 billion of revenue, tourism schools have 48,091 students and total length of routes reaches 60,102 km, much higher than most of other areas. The sustainability of eight areas, including Ningxia (0.142), Qinghai (0.178) and Inner Mongolia (0.204) is extremely low due to the barren and bad ecological environment, and the geographical environment also restricts the external communication and expansion of their tourism industry, thus resulting in the dissatisfying industrial benefit. The sustainability of auxiliary industry of other areas are basically intermediate to low, differing slightly from each other.

**Figure 5:**
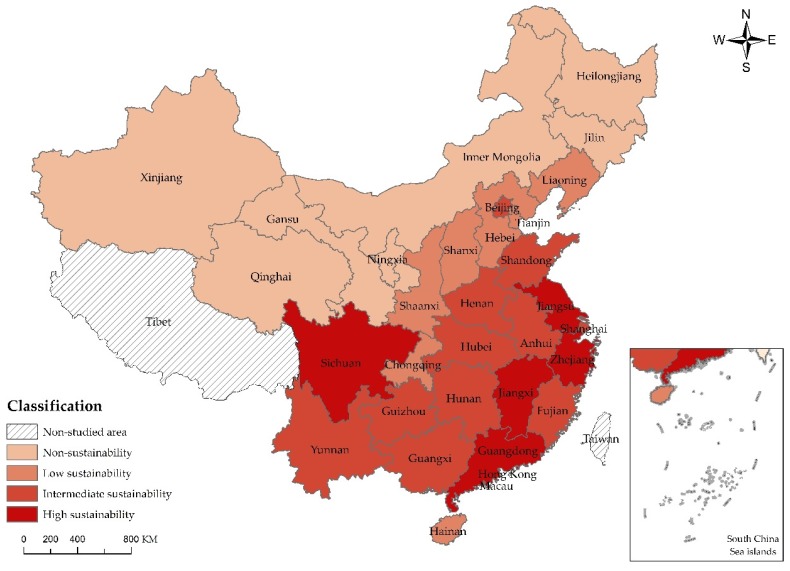
Comparison chart of sustainable capacity of scenic spots in China.

From the overall sustainability of scenic spots (Figure 5), the top three are Guangdong, Jiangsu and Zhejiang. It can be analyzed that such high sustainability comes from the advantageous geographic location, abundant tourism resources, highly developed tourism economy, frequent international exchange and favorable ecological environment. While the sustainability of scenic spots in West China is generally low, Sichuan ranks the 4th due to its abundant tourism resources and superior greening, reasonable resource development, splendid natural landscapes and complete tourism industrial chain. Moreover, under the background of concentrated poverty elimination and tourism-oriented poverty elimination of China, Sichuan implements and enforces support policies of the Chinese government well to keep the overall sustainability of scenic spots much higher than that of other areas in Middle and West China. Beijing, the capital of China, only ranks the 10th because of its disadvantages in the ecological environment and vegetation coverage.

### 6.2. Analysis of Sustainable Capacity of Scenic Spots by Areas

The average value of sustainable capacity of scenic spots in 30 Chinese provinces and cities is 0.444, generally in the intermediate level. The development of Chinese tourism industry differs significantly that it is stronger in East China, intermediate in Middle China and weaker in Northeast and West China. According to the score form, East China is much advantageous to other areas in the scale and benefit of auxiliary industry but is only slightly better than Middle China in the resource condition and ecological environment, so it can be concluded that the sustainable development of scenic spots in East China focuses on the expansion of industrial scale and elevation of economic benefit while concerning the protection and improvement of resources and ecological environment, thus possessing the strong sustainable capacity; moreover, East China covers much coastal areas with obvious geographical advantage and sufficient tourist source. Middle China is full of natural tourism resources and great potentiality in the tourism market (domestic and international tourists are up to 2.692 billion person-times) but behaves ordinary in the scale and benefit of auxiliary industry, and due to the low economic strengthen of tourism and poor geographic condition, its sustainability is quite week. Although Northeast China and West China possess featured landscapes like glacier, snowfield, forest and wetland, the low traffic accessibility, far distance from major tourist sources and weak availability lead to insufficient potentiality in domestic and international tourism market; the extensive development and operation mode restrict the substantial expansion of the scale and benefit of tourism industry; moreover, these areas lack excellent development concepts with respect to the management of scenic spots and improvement of ecological environment; with serious weakness in the service system and tourism infrastructure of scenic spots, the sustainability of Northeast China and West China (0.253 and 0.366) is lower than the average level of China (as shown in Table 9).

## 7. Suggested Countermeasures

In view of the differentiated extensity of sustainable development of Chinese scenic spots and the low sustainable development in West China and Northeast China, following countermeasures are proposed in this paper:

1. For the western and northeastern regions where the sustainable development levels of all aspects of scenic areas are relatively low, the following two measures are proposed:
  (1) Local governments need to increase financial support, improve the scenic area software support to the scenic area function of scientific planning and positioning, give full play to the advantages of scenic resources, create a distinctive tourism brand, improve the attraction and competitiveness of scenic areas, promote the development of the scenic area economy from the policy and technological innovation, and then lead the development of auxiliary industries around the scenic area;
  (2) Developing scenic resources reasonably and paying attention to the protection and management of ecological environment for protecting the future tourism development depends on the existence of environmental quality, improving the scenic area management mode for getting rid of extensive development and management mode of scenic area;
  (3) Improving the basic public facilities and entertainment services around the scenic area, reasonably planning the network of scenic traffic, better the quality of life in the tourist reception area for providing tourists with high quality tourism experience.
2. For the central region where the scale and benefit of the auxiliary industry of scenic spots are not outstanding, it is necessary to excavate the characteristics of its own tourism resources, differentiate and accurately locate the market, and provide high-quality tourism services; Strengthening the cooperation of regional tourism and the comprehensive integration of tourism resources, commonly designing and developing the routes of cross regional tourism, the construction of tourism fine lines and the establishment of barrier-free tourism mechanism, promoting the free flow of industrial factors for accelerating the integration process of regional tourism development.
3. For the eastern region with good scenic resources and ecological management, it is necessary to promote the optimization and upgrading of tourism industrial structure and the accelerated development of tourism economy on the premise of protecting the existing ecological environment. Deepening the reform and innovation of the management system and related policies, constructing a number of tourist pioneer areas and demonstration areas for promoting the development of the central, western and northeastern regions in China, cultivating new growth point of tourism economy innovatively.

## 8. Conclusions

Based on the statistics information of tourism of each province in 2015, this paper establishes the comprehensive measurement index system of the sustainable capacity of scenic spots, further determines the weight of each index by the entropy method, analyzes by TOPSIS method to obtain the comprehensive value of sustainable capacity of scenic spots of each province, and finally conducts the comprehensive analysis of the sustainable capacity of scenic spots of each provinces. Conclusion of this paper is listed below:

(1) After giving comprehensive consideration to the resource condition, economic benefit, auxiliary industrial scale, auxiliary industry benefit and ecological environment in relation to the sustainable capacity of scenic spots, this model establishes a relatively comprehensive sustainable capacity evaluation index system to provide relatively reliable reference to the objective multiple-objective measurement;
(2) Utilizing the principal component analysis and entropy TOPSIS method, this model establishes the sustainable capacity measurement model of scenic spots. Firstly, while decreasing the computation quantity effectively by principal components analysis, this model reduces the multi-dimensional index influencing the sustainable development of scenic spots to the lower dimensional index; secondly, through assigning the objective weight to the lower dimensional index by entropy method, this model avoids the subjective influence brought by the personal preference to make the result more objective and scientific; finally, this model further lowers the weighted low dimensional index to one dimensional index by TOPSIS method to analyze the sustainable development of scenic spots of each province and city in an easier manner. Therefore, this measurement model is better adapted to the sustainable development of scenic spots that it may ensure the scientific and reasonable result while decreasing the workload;
(3) Study on the measurement of the sustainable development of scenic spots is very significant for understanding the sustainable development of scenic spots and their auxiliary industries to establish specific and scientific countermeasures;

Although the measurement index system established in this paper gives comprehensive consideration to indexes in relation to the measurement of sustainable capacity of scenic spots, it does not cover all indexes influencing the sustainable development of scenic spots; therefore, this index system is still restrictive to a certain extent. Besides, principal components analysis may decrease the workload of the large-volume data computation but the converted data volume is still relatively large and the computation is quite complicated. As a result, it is still to be studied as how to better improve the measurement index system and analyze the sustainable development of scenic spots by a faster and more effective measurement method.

## Figures and Tables

**Table 1:** Sustainable Development Measurement Index System of Scenic Spots.

Category	Primary Index	Secondary Index	References
Self-sustainable capacity	Resource condition of scenic spot B1	Number of scenic spots (units) B11	[18,30,34,36,37]
		Area of nature reserves(10,000 hectares) B12	[31]
		Employees of scenic spots (persons) B13	[26] *, [31] *
		Number of domestic tourists (100 million person-times) B14	[15] *, [18], [20] *, [26] *, [29,30,31,32,33,34,35,36,37]
		Number of international tourists (10,000 person-times) B15	[15] *, [18], [20] *, [26] *, [29,30,31,32,33,34,35,36,37]
	Economic benefits of scenic spot B2	Earnings from domestic tourism (100 million yuan) B21	[20,29,30,31,32,34,35,36,37]
		Foreign exchange earnings form international tourism (million dollars) B22	[20,28,29,30,31,32,33,34,35,36,37]
		Revenue of scenic spots (100 million yuan) B23	[34] *
		Contribution of tourism to the tertiary industry (%) B24	[15] *, [20] *, [29,30,32]
		Average stay of tourists (days) B25	[18,27]
	Ecological environment of scenic spot B3	Total emission volume of SO_2_ (ton) B31	[28] *, [29,32]
		Forest coverage rate (%) B32	[15] *, [20,30,34]
		Green covered rate of completed area (%) B33	[18,28,29]
		Centralized pollution control facilities for SO_2_ (unit) B34	[28] *
		Treatment rate of consumption wastes (%) B35	[20,29,30,31,32,34]
		Waste water treatment rate (%) B36	[26,29,31,32,34]
Sustainable capacity of auxiliary industry	Auxiliary industry scale B4	Total travel agencies (unit) B41	[18,28,29,31,35,36]
		Employees of travel agencies (persons) B42	[26] *, [31] *, [36]
		Total number of star hotel beds (unit) B43	[18] *, [27] *, [37]
		Employees of star hotel (persons) B44	[36]
		Number of tourism schools and colleges (schools) B45	[31]
		Number of students at tourism schools and colleges (persons) B46	[18,31,35,37]
		Number of public vehicles under operation (unit) B47	[15] *, [34] *
		Length under operation (km) B48	[15] *, [34] *, [37]
	Auxiliary industrial benefit B5	Revenue of travel agencies (100 million yuan) B51	[31,35,37]
		Revenue of star hotel (100 million yuan) B52	[34] *
		Average room occupancy rate (%) B53	[34] *, [36]
		Passenger turnover (100 million passenger-km) B54	[33]
		Fixed assets revenue per 100 yuan (yuan) B55	[33]
		Total labor productivity (1000 yuan/person) B56	[18,29]

* represents a similar indicator.

**Table 2:** Total variance explained of self-sustainable capacity.

Component	Initial Eigenvalues			Rotation Sums of Squared Loadings		
	Total	% of Variance	Cumulative %	Total	% of Variance	Cumulative %
1	5.769	36.053	36.053	3.513	21.954	21.954
2	2.321	14.505	50.558	3.274	20.463	42.417
3	1.95	12.186	62.744	2.491	15.568	57.985
4	1.727	10.795	73.539	2.016	12.601	70.586
5	1.071	6.692	80.231	1.543	9.646	80.231
6	0.99	6.185	86.416			
L	L	L	L			
15	0.035	0.22	99.809			
16	0.031	0.191	100			

L: Due to the length limitation of the article, L denotes unimportant information and is not displayed here.

**Table 3:** Total variance explained of sustainable capacity of auxiliary industry.

Component	Initial Eigenvalues			Rotation Sums of Squared Loadings		
	Total	% of Variance	Cumulative %	Total	% of Variance	Cumulative %
1	8.607	61.478	61.478	5.287	37.761	37.761
2	2.522	18.011	79.489	5.078	36.271	74.033
3	1.208	8.629	88.118	1.972	14.085	88.118
4	0.501	3.581	91.699			
L	L	L	L			
13	0.015	0.108	99.956			
14	0.006	0.044	100			

L: Due to the length limitation of the article, L denotes unimportant information and is not displayed here.

**Table 4:** Component Score Coefficient Matrix.

Index	Self-Sustainable Capacity					Sustainable Capacity of Auxiliary Industry		
	1	2	3	4	5	1	2	3
B11	−0.079	0.324	−0.018	0.035	−0.184			
B12	0.062	0.09	−0.352	−0.144	0.023			
B13	0.053	0.196	−0.063	0.097	−0.088			
B14	0.078	0.14	0.015	0.015	0.111			
B15	0.347	−0.11	−0.119	−0.033	0.053			
B21	0.155	0.099	0.01	−0.017	0.046			
B22	0.315	−0.126	−0.005	0.025	−0.072			
B23	−0.014	0.108	−0.038	−0.025	0.346			
B24	−0.016	−0.088	−0.001	−0.151	0.7			
B25	0.018	−0.088	0.311	−0.392	−0.025			
B31	0.106	−0.327	0.051	0.15	−0.051			
B32	0.069	−0.186	0.014	0.361	0.133			
B33	−0.027	−0.057	0.399	−0.073	−0.026			
B34	0.246	0.056	−0.03	−0.115	−0.03			
B35	−0.113	0.038	0.119	0.463	−0.282			
B36	−0.087	0.092	0.277	−0.076	0.038			
B41						0.879	−0.151	−0.155
B42						0.933	0.19	−0.198
B43						0.902	−0.091	−0.136
B44						0.95	−0.042	−0.068
B45						0.758	−0.456	0.286
B46						0.788	−0.423	0.273
B47						0.933	−0.188	−0.12
B48						0.885	−0.251	−0.155
B51						0.786	0.518	−0.202
B52						0.891	0.383	−0.174
B53						0.587	0.463	0.531
B54						0.617	−0.599	0.336
B55						0.238	0.565	0.673
B56						0.453	0.812	−0.086

**Table 5:** Decision Matrix and Entropy Weight of Principal Components.

	F1	F2	F3	F4	F5	F6	F7	F8
$w_{j}$	0.102	0.107	0.062	0.079	0.167	0.153	0.167	0.163
Beijing	0.307	−0.921	0.535	0.325	−0.775	3.059	−1.230	0.345
Tianjin	0.387	−1.527	2.748	−3.427	−0.250	−0.352	−0.995	0.498
Hebei	−0.567	0.865	0.070	−0.299	−0.375	−0.123	0.404	−1.414
Shanxi	−0.663	0.111	0.352	−0.510	0.945	−0.182	−0.578	−1.248
Inner Mongolia	−0.817	0.531	−0.318	−0.514	−0.602	−0.021	−0.763	−1.381
Liaoning	−0.027	0.625	0.053	0.240	−0.406	0.296	0.150	−1.134
Jilin	−0.534	−0.817	0.324	0.450	0.082	−0.268	−0.673	−0.984
Heilongjiang	−0.523	−0.101	−0.762	0.876	−1.212	−0.256	−0.535	−0.751
Shanghai	0.120	−0.860	0.470	0.340	−1.311	2.139	−1.573	2.239
Jiangsu	0.285	1.644	0.789	−0.099	−0.671	0.725	1.530	0.903
Zhejiang	0.899	0.246	0.407	0.939	−0.039	1.394	0.979	0.095
Anhui	−0.389	0.544	0.661	0.253	0.646	−0.591	0.652	−0.164
Fujian	0.169	−1.149	0.725	1.299	−0.458	−0.126	−0.068	1.848
Jiangxi	−0.334	−0.045	0.394	0.526	2.565	−0.746	−0.044	0.678
Shandong	0.164	3.273	0.422	−0.174	−1.190	0.869	1.358	−0.662
Henan	−0.443	0.930	0.088	−0.138	−0.070	−1.235	1.360	0.577
Hubei	−0.165	0.231	0.027	0.263	0.264	−0.524	0.786	0.150
Hunan	−0.139	0.675	0.099	1.277	−0.256	−0.808	0.885	1.341
Guangdong	4.862	−0.117	−0.420	−0.057	0.170	2.168	2.330	−1.183
Guangxi	−0.038	−0.575	0.008	0.861	0.639	−0.831	0.338	0.303
Hainan	−0.328	−1.742	−0.534	1.470	−0.710	−0.254	−1.166	0.949
Chongqing	−0.434	−0.419	0.768	0.372	−0.304	−0.642	0.008	0.945
Sichuan	0.384	0.991	−0.637	−0.023	1.460	−0.654	0.891	0.519
Guizhou	−0.572	−0.264	−0.105	−0.191	2.564	−0.988	−0.220	0.753
Yunnan	0.129	−0.430	−0.333	0.209	1.222	−0.365	0.584	−0.672
Shaanxi	−0.403	−0.033	0.454	0.419	0.191	−0.374	−0.026	0.307
Gansu	−0.242	−0.084	−1.911	−1.823	0.554	−0.521	−0.725	−0.262
Qinghai	0.019	−0.952	−3.237	−0.693	−0.728	−0.422	−1.389	−0.675
Ningxia	−0.703	−0.861	0.255	−0.290	−1.402	−0.131	−1.483	−1.663
Xinjiang	−0.402	0.234	−1.394	−1.881	−0.543	−0.236	−0.786	−0.258

**Table 6:** Ideal solution.

$Z^{+}$	0.496	0.350	0.170	0.116	0.429	0.467	0.390	0.365
$Z^{-}$	−0.083	−0.186	−0.200	−0.270	−0.235	−0.189	−0.263	−0.271

**Table 7:** Sustainable capacity value of scenic spots of 30 Chinese provinces and cities.

Region	Self-Sustainable Capacity of Scenic Spot		Sustainable Capacity of Auxiliary Industry		Sustainable Capacity of Scenic Spot	
	Value	Rank	Value	Rank	Value	Rank
Beijing	0.324	22	0.523	5	0.435	10
Tianjin	0.322	23	0.324	21	0.323	23
Hebei	0.360	18	0.310	22	0.337	21
Shanxi	0.423	9	0.212	26	0.338	20
Inner Mongolia	0.315	24	0.204	28	0.269	27
Liaoning	0.376	16	0.327	20	0.354	19
Jilin	0.357	19	0.212	27	0.299	24
Heilongjiang	0.306	26	0.243	25	0.281	26
Shanghai	0.298	27	0.550	4	0.442	9
Jiangsu	0.416	11	0.625	1	0.509	2
Zhejiang	0.440	8	0.572	3	0.501	3
Anhui	0.444	6	0.382	14	0.416	13
Fujian	0.364	17	0.505	6	0.433	11
Jiangxi	0.545	2	0.383	13	0.479	5
Shandong	0.442	7	0.503	7	0.467	6
Henan	0.390	15	0.457	9	0.426	12
Hubei	0.402	13	0.421	11	0.411	14
Hunan	0.412	12	0.495	8	0.454	8
Guangdong	0.555	1	0.594	2	0.576	1
Guangxi	0.421	10	0.378	15	0.401	16
Hainan	0.315	25	0.363	18	0.337	22
Chongqing	0.352	20	0.415	12	0.382	18
Sichuan	0.521	3	0.449	10	0.486	4
Guizhou	0.516	4	0.366	17	0.454	7
Yunnan	0.454	5	0.353	19	0.408	15
Shaanxi	0.393	14	0.373	16	0.384	17
Gansu	0.333	21	0.254	24	0.297	25
Qinghai	0.220	30	0.178	29	0.202	30
Ningxia	0.254	29	0.142	30	0.210	29
Xinjiang	0.256	28	0.268	23	0.262	28

**Table 8:** The division basis and explanation of the sustainable development of scenic spots.

Rank	Range	Classification	Explanation
Level 1	>0.4537	High sustainability	Scenic resources development scientific planning, high degree of governance for ecological environment, scenic auxiliary industry development is highly mature
Level 2	0.3926–0.4536	Intermediate sustainability	Scenic resources development is reasonable, the effective management of ecological environment, scenic auxiliary industry reached a certain scale but the benefits are not outstanding
Level 3	0.3239–0.3925	Low sustainability	The pattern of scenic resources development needs to be improved, the attention of ecological management is not enough, the scale and benefit of scenic auxiliary industry are not significant
Level 4	0–0.3238	Non-sustainability (unacceptable)	The exploitation of scenic resources is extremely unreasonable, the pollution of ecological environment is serious, the auxiliary industry is not large and the benefit is low

**Table 9:** Comparison of sustainable development ability of four regional scenic spots.

Region	Self-Sustainable Capacity of Scenic Spot		Sustainable Capacity of Auxiliary Industry		Sustainable Capacity of Scenic Spot	
	Value	Rank	Value	Rank	Value	Rank
East	0.608	1	0.933	1	0.712	1
Central	0.558	2	0.331	2	0.446	2
West	0.344	4	0.044	4	0.253	4
Northeast	0.422	3	0.307	3	0.366	3
National average	0.483	-	0.404	-	0.444	-
